# Effects of a diverse prebiotic fibre blend on inflammation, the gut microbiota and affective symptoms in metabolic syndrome: a pilot open-label randomised controlled trial

**DOI:** 10.1017/S0007114524002186

**Published:** 2024-10-28

**Authors:** Caitlin Victoria Hall, Piril Hepsomali, Boushra Dalile, Leonardo Scapozza, Thomas Gurry

**Affiliations:** 1 Myota Limited, London, UK; 2 School of Psychology and Clinical Language Sciences, University of Reading, Reading, UK; 3 Translational Research Center in Gastrointestinal Disorders (TARGID), Department of Chronic Diseases and Metabolism, Faculty of Medicine, KU Leuven, Leuven, Belgium; 4 Leuven Brain Institute, KU Leuven, Leuven, Belgium; 5 Laboratory of Biological Psychology, Brain & Cognition, Faculty of Psychology and Educational Sciences, KU Leuven, Leuven, Belgium; 6 Pharmaceutical Biochemistry Group, School of Pharmaceutical Sciences, University of Geneva, Geneva, Switzerland

**Keywords:** Microbiota–gut-brain axis, Depression, Anxiety, Stress, High sensitivity C-reactive protein, Inflammation, Prebiotic fibre

## Abstract

Emerging evidence suggests that low-grade systemic inflammation plays a key role in altering brain activity, behaviour and affect. Modulation of the gut microbiota using prebiotic fibre offers a potential therapeutic tool to regulate inflammation, mediated via the production of short-chain fatty acids (SCFA). However, the impact of prebiotic consumption on affective symptoms and the possible contribution from inflammation, gut symptoms and the gut microbiome are currently underexamined. In this 12-week study, the effects of a diverse prebiotic blend on inflammation, gut microbiota profiles and affective symptoms in a population with metabolic syndrome (MetS) were examined. Sixty males and females with MetS meeting the criteria for MetS were randomised into a treatment group (*n* 40), receiving 10 g per day of a diverse prebiotic blend and healthy eating advice, and a control group (*n* 20), receiving healthy eating advice only. Our results showed a significant reduction in high sensitivity C-reactive protein (hs-CRP) in the treatment (–0·58 [–9·96 to–2·63]) compared with control (0·37 [–3·64 to–3·32]), alongside significant improvements in self-reported affective scores in the treatment compared with the control group. While there were no differences in relative abundance between groups at week 12, there was a significant increase from baseline to week 12 in fecal *Bifidobacterium* and *Parabacteroides* in the treatment group, both of which are recognised as SCFA producers. Multivariate regression analyses further revealed an association between gastrointestinal symptoms and hs-CRP with affective scores. Together, this study provides preliminary support for a diverse prebiotic blend for mood, stress and anxiety.

Metabolic syndrome (MetS) is defined as a cluster of risk factors including visceral or central obesity, glucose dysregulation, hypertension and dyslipidaemia^(1)^. Alongside metabolic abnormalities, MetS is strongly associated with low-grade systemic inflammation, which is thought to play a critical role in the development of mood, stress, anxiety and sleep disorders and/or symptomology^(2–5)^. As such, there is a strong bidirectional relationship between MetS, with anxiety disorders^(4)^ and major depression^(6)^, with population-based studies showing an increased prevalence of anxiety and depression among individuals with MetS^(7)^. As part of this underlying mechanism, pro-inflammatory cytokines communicate with the central nervous system by activating receptors on vagal afferents or by generating intermediates at the blood–brain barrier interface^(8)^. The brain recognises and interprets inflammation as a signal of illness, which collectively gives rise to ‘sickness behaviours’, including social avoidance, anhedonia, fatigue, anxiety and depressed mood^(9–11)^. While sickness behaviours were initially studied in the context of acute inflammation (i.e. infection), more recent work shows that repeated or long-term exposure to low-grade systemic inflammation, as seen in conditions like MetS, is associated with an increased risk of future depressive and anxiety symptoms^(11–14)^. There is, therefore, a growing interest to develop novel, simple and cost-effective interventions that can target systemic inflammation to improve long-term psychological health.

Recent work suggests that the gut microbiota and its metabolites can have a significant effect on attenuating low-grade systemic inflammation^(15)^, offering a potential therapeutic avenue to improve affective symptoms in MetS. One of the primary mechanisms by which the gut microbiota influences inflammation is via the production of short-chain fatty acids (SCFA) during the microbial fermentation of prebiotic fibre^(16)^. SCFA interact with virtually all pathways mediating gut–brain communication, including neuronal (vagal nerve), humoral, endocrine and immune pathways^(17)^. Specifically, SCFA can affect the central nervous system by regulating inflammation both locally^(18)^ and at the blood–brain barrier^(8,14)^, rapidly signalling to the brain via enteroendocrine-mediated vagal signalling^(19,20)^, or affecting gene expression by acting as histone deacetylase inhibitors (HDAC)^(21)^. SCF have also been shown to modulate blood lipids and blood pressure, which beyond being a central characteristic in MetS have been shown to influence brain function through both peripheral and central mechanisms, including the reduction of peripheral atherogenic and proinflammatory effects^(22)^. Beyond its effects on the gut microbiota, soluble fibre directly impacts lipid profiles and blood pressure related to its viscous properties^(23)^. To leverage the proposed benefits of SCFA, prebiotic supplements have been utilised to investigate effects on mood, anxiety, stress and cognition^(24–30)^. Importantly, these studies have been performed in sub-clinical or healthy populations, suggesting that the benefits of prebiotic interventions extend beyond major neuropsychiatric conditions. While early findings are promising, they often fail to assess broader gastrointestinal (GI), inflammatory, and microbial changes that may parse psychological improvements. In this study, we assessed the effects of a diverse prebiotic blend on systemic inflammation, GI symptoms, self-reported affective scores, and the gut microbiota, in a population with MetS.

## Methods

### Participants

Eligible participants were males and females (aged 18–75 years) who met the International Diabetes Federation criteria for MetS^(1)^, yet were not receiving treatment for their symptoms. MetS is defined as having abdominal obesity (waist circumference ≥ 94 cm in men, and ≥ 80 cm in women) plus two or more of the following: raised TAG (≥ 1·7 mmol/l); reduced HDL-cholesterol (< 1·03 mmol/l in men and < 1·29 mmol/l in women); raised systolic blood pressure (≥ 130 mmHg); raised diastolic blood pressure (≥ 85 mmHg); treatment of previously diagnosed hypertension; raised fasting plasma glucose (≥ 5·6 mmol/l) or previously diagnosed with pre-diabetes. Main exclusion criteria included a current diagnosis of Type 1 or 2 diabetes or cardiovascular disease, or receiving medications that lower cholesterol, blood pressure, or blood glucose levels. Detailed inclusion and exclusion criteria are reported in online Supplementary Note 1.

### Study design

The study (conducted in the UK between 19 June 2023 and 22 December 2023) was a 12-week open-label parallel randomised controlled trial, with two groups: a control group who received healthy eating advice and a treatment group who received healthy eating advice and consumed a prebiotic fibre supplement (10 g/d) (online Supplementary Note 2). The study was a decentralised design, where participants completed all study requirements via video call and self-administered sample collection kits. Study requirements were completed during a pre-intervention baseline session (baseline) and a follow-up session at the end of the 12-week intervention (week 12). All participants had a video call with a member of the research team prior to the baseline session to confirm they understood the instructions for completing questionnaires, sample collection and daily prebiotic intake. At the end of the study, participants were encouraged to continue healthy eating and lifestyle changes recommended to them during the study. Additionally, if a participant’s lipid or hs-CRP results had worsened since their baseline assessment, which warranted additional intervention (i.e. medication or specialist care), the participants were advised to contact their personal GP or healthcare provider to seek further advice.

### Randomisation

The sequence of random allocation was generated using custom MATLAB code for stratified block randomisation. Stratification was performed according to sex and age. An independent investigator, who was not involved in the assessment of participants or in data collection and analysis, performed the randomisation for all participants. Each eligible participant was assigned to the Treatment (*n* 40) or Control (*n* 20) groups in a 2:1 ratio using permuted blocks within each stratum. This prebiotic blend has not been studied formally in a cohort of participants with MetS. Therefore, a 2:1 treatment allocation was adopted to yield more information about potential tolerance and side effects in MetS, while controlling cohort size for practical purposes.

### Prebiotic treatment

The prebiotic blend was powdered, unflavoured and given to the participants in 300 g packets lasting for 30 d each (total three packets per participant). The blend included the following ingredients: fructooligosaccharides, inulin, resistant dextrin, resistant maltodextrin, partially hydrolysed guar gum, and guar gum (myota prebiotic blend). The dose of each ingredient was selected based on earlier evidence supporting their ability to reliably produce SCFA in the gut microbiota across populations^(31)^ and previous work demonstrating their anti-inflammatory effects using a comparable blend of prebiotic ingredients^(32)^ (online Supplementary Note 2). A 10 g scoop was included in each packet, and participants were advised to consume one level scoop at any time of the day. Participants were provided with examples on how to consume the prebiotic blend (e.g. breakfast cereal, coffee, tea and water). To minimise participant withdrawal and ensure consistent use, they received a weekly survey via email, asking them to confirm that they had taken the prebiotic blend on each day of the week. If they failed to consume the prebiotic on any given day, they were prompted to provide a reason.

### Healthy eating advice

All participants were provided with healthy eating advice prior to starting the 12-week intervention. Dietary recommendations were available via the Thriva online portal and were consistent with the Heart UK’s Healthy Eating Guidelines for MetS (online Supplementary Note 3). These recommendations emphasised a Mediterranean diet, rich in fruit, vegetables and healthy fats (*n*-3 fatty acids), while reducing refined sugar, salt, processed foods and alcohol intake. The Mediterranean diet is considered gold-standard dietary approach for participants with MetS and/or poor mental health^(33,34)^.

### Questionnaires

Participants completed previously validated questionnaires including the 18-item FiberScreen^(35)^, Gastrointestinal Symptom Rating Scale (GSRS)^(36)^, Perceived Stress Scale (PSS)^(37)^, Depression Anxiety and Stress Scale (42-item) (DASS)^(38)^, Patient Health Questionnaire-9^(39)^ and Generalised Anxiety Disorder (7-item)^(40)^ at both timepoints.

To assess the effect of the intervention on affective scores, we assessed changes in the DASS and PSS scales. The DASS is a 42-item self-report instrument that is based on a dimensional, rather than categorical, assessment of psychological symptoms, providing high inter-subject variability in non-clinical and sub-clinical populations^(41)^. Critically, the DASS can clearly distinguish between the three negative affective states of depression, anxiety, and stress^(42)^. The PSS is a 10-item self-report instrument which asks participants how different situations affect feelings and perceived stress in the previous month^(37)^. This scale has also been validated in sub-clinical populations and also provides high inter-individual variability^(43)^.

The Patient Health Questionnaire-9 and Generalised Anxiety Disorder (7-item) are clinical tools that are used for screening, diagnosing and monitoring depression and generalised anxiety disorders, respectively. In this study, these assessments were performed to (a) rule out severe neuropsychiatric disorders at baseline and (b) assess the development of major neuropsychiatric disorders during the intervention which may require withdrawal from the study and/or ongoing monitoring from the participant’s primary care team.

### Blood sample collection and processing

Finger-prick capillary blood sampling kits (Thriva Limited, UK) were shipped to the participant’s chosen address, with written instructions on correct usage. Blood samples (600 ul) were collected after an 8-hour minimum fast. Participants were advised to return their sample immediately after collection using a prepaid envelope. Once samples arrived at the lab, Serum Separator Tubes (SST) were centrifuged to separate the blood serum within the sample for testing. This occurs immediately after arriving at the lab to prevent sample decay. The samples are then processed within a 48-hour window. Hs-CRP and lipid profiles (LDL-cholesterol, HDL-cholesterol, total cholesterol and TAG) were tested via a Roche Cobas c503 platform. Previous work has shown high intraclass correlation coefficients between self-sampling of capillary blood compared with venous analysis, as well as high patient tolerability^(44,45)^. In the event where a participant’s blood sample could not be analysed due to insufficient sample volume, a replacement kit was sent to the participant. At baseline, this involved delaying the participant’s start time until a sufficient sample was obtained. At the 12-week follow-up, if the second attempt resulted in an insufficient sample, the participant was excluded from the hs-CRP and lipid analysis.

### Anthropometric and blood pressure measures

Height and waist and hip circumference measurements were taken by the participant with a measuring tape provided in the postal pack. Written and visual instructions were provided to each participant with guidelines on how to record accurate measurements. Participants were asked to weigh themselves and take a blood pressure reading using either a home weighing scale or blood pressure monitor or visiting a local pharmacy. Participants were asked to take their blood pressure after sitting down for at least 5–10 min. Participants were advised to use the same measuring devices for both baseline and week 12 measurements.

### Stool sample collection and 16S rRNA gene sequencing

Participants were provided with a stool sampling kit (Carbiotix AB (publ), Lund, Sweden), including a sample collection tube, sample swab and instruction card to use at home. Participants were instructed to collect the sample within 24 h of completing other study requirements (blood sample and questionnaires) at baseline and week 12. The sample collection tube contained a buffer solution which stabilises RNA and DNA in samples at ambient temperatures, and therefore does not require freezing prior to sequencing. Each stool sample was labelled, and an aliquot was used directly for sequencing while the remainder was stored in a − 80°C freezer. DNA was extracted from the stool samples via homogenisation (3000 rpm, 2 min) prior to the removal of 200 ul of slurry to be subjected to NA extraction on the kingfisher flex 96 system from Thermo Fisher Scientific. q-PCR amplification was conducted with 40 cycles of 95°C denaturing, 55–60°C annealing and 72°C extension steps with Dual-Lock^(TM)^ DNA polymerase. Primers targeting the V4 (515F, 806R) region of the 16S gene were used. 16S rRNA gene sequencing was performed on an Illumina MiSeq platform^(46,47)^.

### 16S rRNA gene sequencing data processing and analysis

Demultiplexed FASTQ files were processed using QIIME2 2020·2 (https://qiime2.org)^(48)^. Reads were quality filtered with a cut-off quality score of 20 and trimmed to a length of 150. Operational taxonomic units were generated by denoising with Deblur^(49)^. For taxonomic structure analysis, taxonomy was assigned to operational taxonomic units using a pre-trained Naïve Bayes classifier and the q2-feature-classifier plugin against the GreenGenes (gg_13_5) 16S rRNA gene sequencing database^(50)^. Samples were rarefied to a read depth of 10 000 for diversity analyses. Wilcoxon signed-rank tests was used to test for group differences in Shannon diversity and Chao1 richness measures. Beta-diversity, assessed using Bray Curtis distance, was used to compare between-group and within-group differences using Permutational Multivariate Analysis of Variance test (ADONIS2). Microbiome Multivariable Associations with Linear Models 2 (MaAsLin2)^(51)^ was used to assess (A) the changes in microbial abundance (collapsed at genus level) from baseline to week 12 in the treatment arm; and (B) the changes in microbial abundance (collapsed at genus level) between the treatment and control arm at week 12. Covariates, including sex, age, and BMI, were included as fixed effects, and participant ID was included as a random effect (for model A only). Given the putative role of the gut microbiota in modulating immune responses, we also performed an exploratory analysis assessing the relationship between hs-CRP and microbiota features. To do this, we included intervention (group), time, and hs-CRP as fixed effects, and participant ID as a random effect. For all models, features were included if they had at least 10 % non-zero values (across samples) and a minimum relative abundance threshold of 0·0001, both validated parameter settings in MaAsLin2. Significant features with *P* < 0·05 and *q* < 0·25 were considered statistically significant.

### Primary and secondary endpoints

The primary endpoint was the change in hs-CRP from baseline to week 12. Key secondary endpoints included changes in DASS-S, DASS-D, DASS-A, PSS, gut microbiome profiles, lipid profiles, anthropometry (weight, BMI, waist, and hip circumference) and systolic and diastolic blood pressure from baseline to week 12.

### Tolerability and safety

Participants were asked to record whether they experienced any side-effects or medical events since starting the intervention, recorded via online surveys at the end of weeks 4, 8, and 12. If they answered yes, severe adverse events and adverse events were documented, reported, and reviewed for relatedness and expectedness within 24 h. Importantly for our primary endpoint, participants were instructed to report any potential sickness or infection symptoms during the study. Research staff monitored baseline and week 12 blood test results and escalated any findings suggestive of infection or acute inflammatory responses. Participants with elevated hs-CRP levels consistent with an infection were given the option to defer participation and repeat baseline tests. If infection was confirmed during the study, affected participants were excluded from the final hs-CRP analysis to prevent confounding effects.

### Statistical tests

Data were analysed using R software (V4.3.1). Statistical analyses were performed based on an intention-to-treat analysis. Group differences in baseline characteristics were assessed using χ2-tests for categorical variables, and parametric *t* tests or Wilcoxon signed-rank tests for quantitative variables. For all comparisons, we tested the normality and homoscedasticity. For the primary and secondary endpoints, an unpaired two-tailed *t* test was used to compare the change from baseline to week 12 (i.e. week 12 – baseline) between the treatment and control groups. The choice to use unpaired *t* tests was adopted to mitigate the influence of baseline differences and to ensure robustness to unequal sample sizes. As an exploratory analysis, stepwise multiple linear regression models were performed to estimate the association between changes in gastrointestinal symptoms and hs-CRP with changes in affective symptoms. The variable hs-CRP was square root transformed prior to analyses. The models combined both groups (control and treatment) and included a regressor which adjusted for the effects of repeated measures. To confirm that multiple regression results remained consistent, we performed a sensitivity analysis which adjusted for the effects age, sex, and BMI (online Supplementary Table 2) and when removing outliers (online Supplementary Table 3). A *P*-value < 0·05 was considered statistically significant. As a pilot study, our sample size was informed based on previous work assessing changes in our primary outcome, hs-CRP, following a prebiotic or dietary intervention in a similar population. Results from previous studies in MetS (*n* 52^(52)^; *n* 50^(53)^), pre-diabetes (*n* 51^(54)^), and non-alcoholic fatty liver (*n* 66^(55)^) suggest that > 50 participants is an adequate sample size to capture meaningful differences in our outcome measures. For feasibility purposes, a sample of sixty participants with MetS, with overrepresentation in the treatment relative to control group (2:1), was chosen.

## Results

### Baseline patient characteristics

**Fig. 1.**
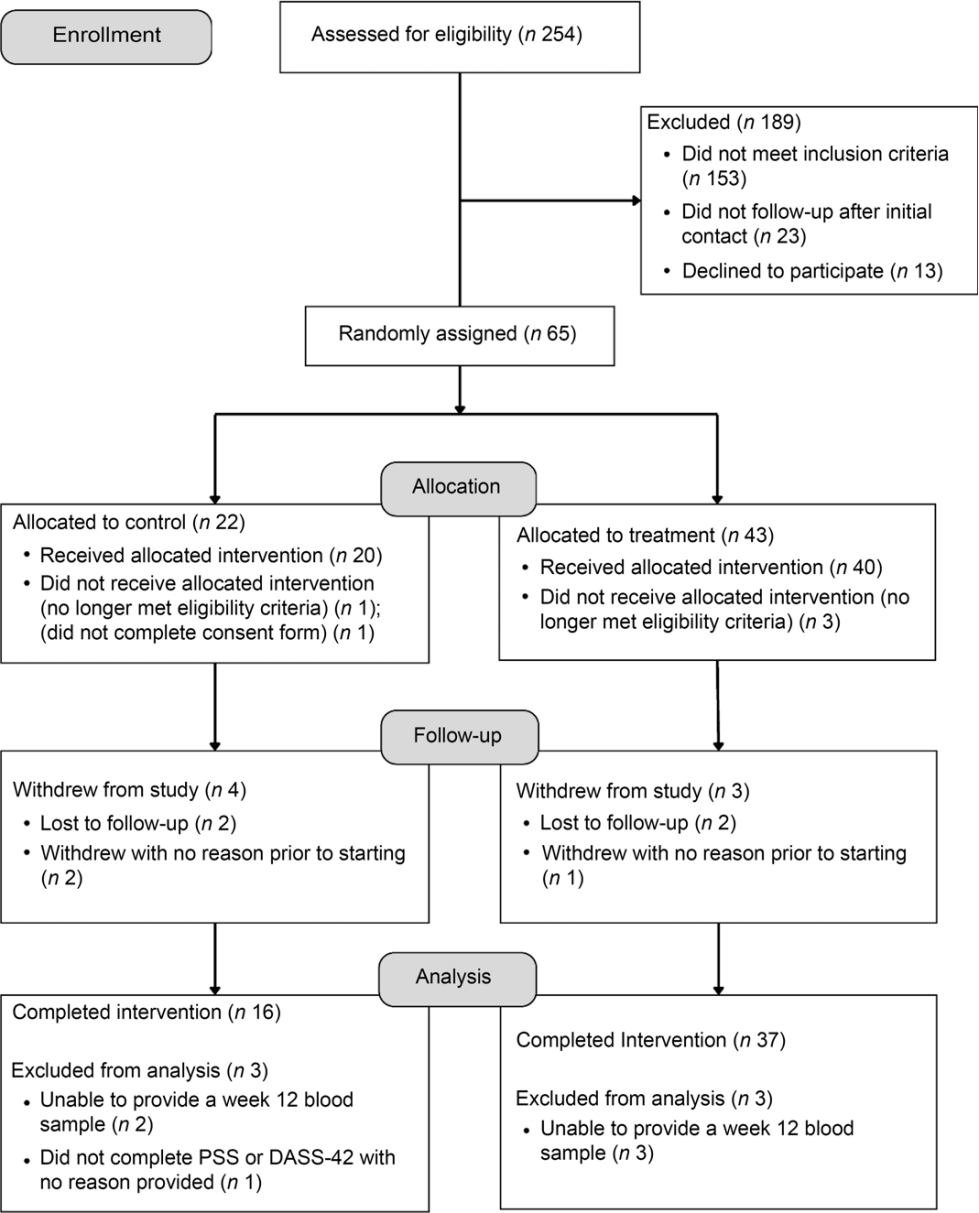
Study participant flow diagram showing participant recruitment and withdrawals from the treatment and control groups.

In total, sixty participants (53·9 (9·8)) years (mean (sd)); 62 % female) who met the International Diabetes Federation criteria for MetS^(1)^ and passed the screening and eligibility assessments were randomised to either the treatment (*n* 40) or control (*n* 20) group, stratified by age and sex (Table 1). Groups were matched except for hip circumference, which was not a matched variable for inclusion or randomisation (Table 1). A total of fifty-three participants (thirty-seven treatment; sixteen control) completed the baseline and week twelve assessments. Reasons for participant dropouts included: lost to follow-up (two treatment; two control) and withdrawal with no reason provided prior to starting the intervention (one treatment; two control) (Fig. 1).

**Table 1.** Baseline participant demographics (Mean values and standard deviations; numbers and percentages)

Demographics	Treatment (*n* 37)	Control (*n* 16)	*P*-value
Gender	27 female (67.5%)	9 female (56%)	0·38*(X^2^ = 0·77)
Age (years)	53·6 ± 10·1	53·9 ± 9·3	0·90^†^
BMI (kg/m^2^)	33·9 ± 13·4	28·9 ± 3·7	0·38^†^
Waist circumference (cm)	103·0 ± 15·0	97·8 ± 7·2	0·24^†^
Hip circumference (cm)	113·7 ± 13·9	105·4 ± 11·1	**0·05** ^**†**^
Current smokers	4 (10·8 %)	2 (12·5 %)	1·0*(X^2^ = 6·7 × 10^–32^)
Pre-diabetes diagnosis	13 (35·1 %)	8 (50 %)	0·48*(X^2^ = 0·50)
Daily fibre intake (g)	17·2 ± 6·9	15·0 ± 4·8	0·29^†^
GAD-7	6·7 ± 5·4	8·3 ± 5·6	0·36^†^
PHQ-9	7·5 ± 6·1	9·3 ± 7·0	0·37^†^

Mean ± sd; total number (percentage); *Pearson’s Chi-squared test; ^†^Parametric *t* test. GAD-7, Generalised Anxiety Disorder 7-item scale; PHQ-9, Patient Health Questionnaire-9.

### Adherence, tolerability and safety

On average, participants in the treatment group consumed the prebiotic blend 73·0 (28·5) % (mean ± sd) of the total intervention time. The treatment was well tolerated, with a larger reduction in total GSRS scores reported in the treatment (–0·34 ± 0·61) compared with the control (–0·06 ± 0·41), although not reaching statistical significance (*t*_*31·37*_ = −1·84, *P* = 0·08, *d* = 0·50). When looking at the subscales of the GSRS, we only observed a significant reduction in abdominal pain in the treatment group (–0·41 ± 0·63) compared with the control (0·23 ± 0·53) (*t*_*24·59*_ = −3·52, *P* = 0·002, *d* = 0·30) (Table 2). There were no severe adverse events related to the treatment, and no participants withdrawing from the study due to product-related side-effects or disliking the taste. Adverse events reported were consistent with those typically seen in prebiotic-based interventions, including wind (*n* 19), bloating (*n* 9), mild constipation (*n* 7), abdominal pain (*n* 6), mild diarrhoea (*n* 5) and burping (*n* 1).

**Table 2.** Baseline, week 12 and delta (week 12 – Baseline) results for clinical, anthropometric and affective outcomes in the treatment and control groups (Mean values and standard deviations; median values and ranges)

	Treatment						Control						
	Baseline		Week 12		Delta		Baseline		Week 12		Delta		
	Mean	sd	Mean	sd	Mean	sd	Mean	sd	Mean	sd	Mean	sd	*P*-value
Hs-CRP (mg/l)													
Median	1·41		1·82		–0·58		1·28		2·36		0·37		0·03*
Range	0–14·8		0·15–13·3		–9·96–2·63		0·15–30·6		0·15–32·6		–3·64–3·32		
PSS	17·85	6·67	13·34	5·83	–4·43	4·76	19·36	5·68	19·00	5·26	–0·54	3·45	**0·004** ^†^
DASS-D	7·28	9·34	4·00	4·49	–3·30	8·04	13·21	13·89	11·14	13·13	0·08	3·37	**0·05** ^†^
DASS-A	5·25	5·71	2·71	3·47	–2·32	4·60	8·64	9·68	7·43	9·22	0·00	2·30	**0·03** ^†^
DASS-S	12·28	9·96	7·08	7·81	–5·14	7·09	13·43	11·83	11·36	10·80	–0·17	4·61	**0·009** ^†^
GAD-7	6·72	5·39	3·87	3·49	–2·70	3·93	8·29	5·55	8·13	5·44	–0·15	3·29	**0·03** ^†^
PHQ-9	7·50	6·14	4·55	3·90	–2·92	4·86	9·29	7·02	8·20	5·71	–1·23	4·71	0·28^†^
GSRS-Total	1·85	0·58	1·54	0·44	–0·34	0·61	2·33	1·14	2·21	1·02	–0·06	0·41	0·08^†^
GSRS-Reflux	1·42	0·83	1·36	0·77	–0·09	0·83	1·96	1·54	1·93	1·27	0·00	1·04	0·77^†^
GSRS-Indigestion	2·38	0·92	2·02	0·91	–0·41	1·09	2·93	1·79	2·57	1·38	0·16	0·65	0·70^†^
GSRS-Pain	1·70	0·63	1·32	0·43	–0·41	0·63	1·76	0·73	1·89	0·77	0·23	0·53	**0·002** ^†^
GSRS-Constipation	2·17	1·59	1·67	0·76	–0·52	1·67	2·64	1·22	2·42	1·10	–0·15	1·34	0·43^†^
GSRS-Diarrhoea	1·56	0·72	1·32	0·51	–0·26	0·63	2·33	1·88	2·22	1·73	–0·05	0·64	0·32^†^
Body weight (kg)	88·9	20·35	86·67	19·6	–1·15	2·94	84·89	14·35	84·27	13·72	–0·88	2·14	0·72^†^
BMI (kg/m^2^)	33·86	13·37	30·65	6·16	–1·64	6·28	29·60	4·04	28·92	3·74	–0·57	0·93	0·32^†^
Waist circumference (cm)	103·00	15·00	99·21	14·79	–3·08	5·86	97·97	6·96	95·43	7·19	–2·39	3·87	0·63^†^
Hip circumference (cm)	113·73	13·87	110·41	15·15	–3·01	10·13	104·98	10·77	101·59	11·29	–3·69	9·41	0·83^†^
Waist:hip ratio (W:H)	0·91	0·09	0·90	0·08	–0·01	0·06	0·94	0·08	0·95	0·09	0·01	0·07	0·47^†^
Systolic BP (mmHg)	133	14·8	126	17·5	–7·19	19·4	128	11·4	130	14·0	2·23	10·1	**0·03** ^†^
Diastolic BP (mmHg)	84·0	7·5	83·3	12·5	–0·28	13·1	81·1	10·5	82·8	8·30	0·46	6·13	0·79^†^
Cholesterol (total) (mmol/l)	5·95	0·96	5·81	1·16	–0·05	0·62	5·48	1·27	5·50	0·97	–0·18	1·17	0·72^†^
TAG (mmol/l)	2·00	1·19	1·82	0·84	–0·07	0·50	1·94	1·14	1·61	0·72	–0·40	0·62	0·11^†^
LDL-cholesterol (mmol/l)	3·71	0·90	3·63	0·97	–0·01	0·60	3·34	1·28	3·38	0·85	–0·15	1·10	0·67^†^
HDL-cholesterol (mmol/l)	1·43	0·41	1·36	0·34	–0·09	0·21	1·44	0·42	1·38	0·46	–0·06	1·81	0·62^†^

Mean (sd); Median (range); *Mann–Whitney U test; ^†^Unpaired *t* test. BP, blood pressure; DASS, Depression, Anxiety, and Stress 42-item scale; DASS-D, (DASS)-Depression; DASS-A, DASS-anxiety; DASS-S, DASS-stress; GSRS, Gastrointestinal Symptom Rating Scale; hs-CRP, high sensitivity C-reactive protein; PSS, Perceived Stress Scale.

### Dietary fibre intake

At baseline, dietary fibre intake (g/d) was comparable between the treatment (17·2 ± 6·9) and control (15·0 ±4·8) groups (mean ± sd) (Table 1). While dietary advice provided to participants emphasised the importance of a plant-based diet, we observed no significant change in dietary fibre intake (g/d) from baseline to week 12 between the treatment (0·82 ±5·57) and control (–0·33 ± 5·57) groups (*t*_*21·07*_ = −0·64, *P* = 0·53, *d* = 0·21) (excluding the prebiotic blend for the treatment group).

### Primary endpoint

**Fig. 2.**
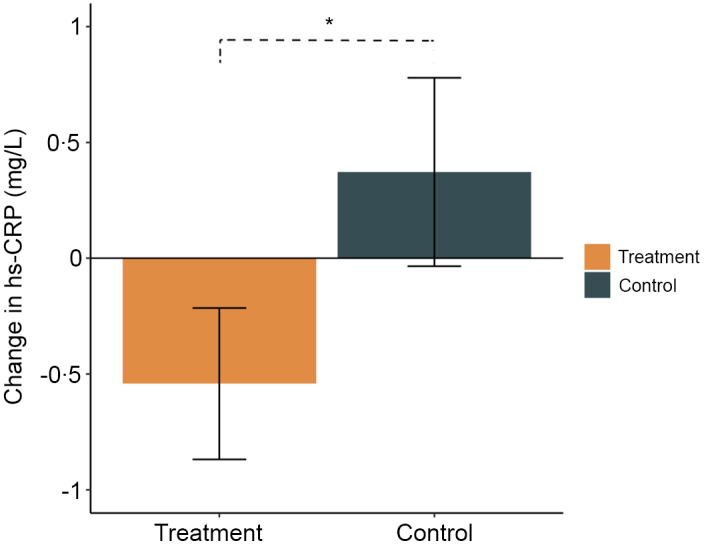
Changes from baseline to week 12 in high sensitivity C-reactive protein (hs-CRP) (mg/l) in the treatment (orange) and control (green) groups. Mean and standard errors are shown. *indicates *P* value < 0·05.

Baseline hs-CRP levels reflected a considerable degree of variability across all participants (1·41 mg/l (0–30·6), median (range)) (Table 2). The change in hs-CRP (mg/l) from baseline to week 12 was significantly different between the treatment (–0·58 [–9·96 to –2·63]) and control groups (0·37 [–3·64 to –3·32]) (*W* = 98, *P* = 0·03, *HLE* = 0·43) (Fig. 2; Table 2). We also performed a sensitivity analysis after removing two outliers and showed consistent results (*W* = 75, *P* = 0·01, *HLE* = 0·51) (online Supplementary Fig. 1(a)–(b)).

### Affective scores

**Fig. 3.**
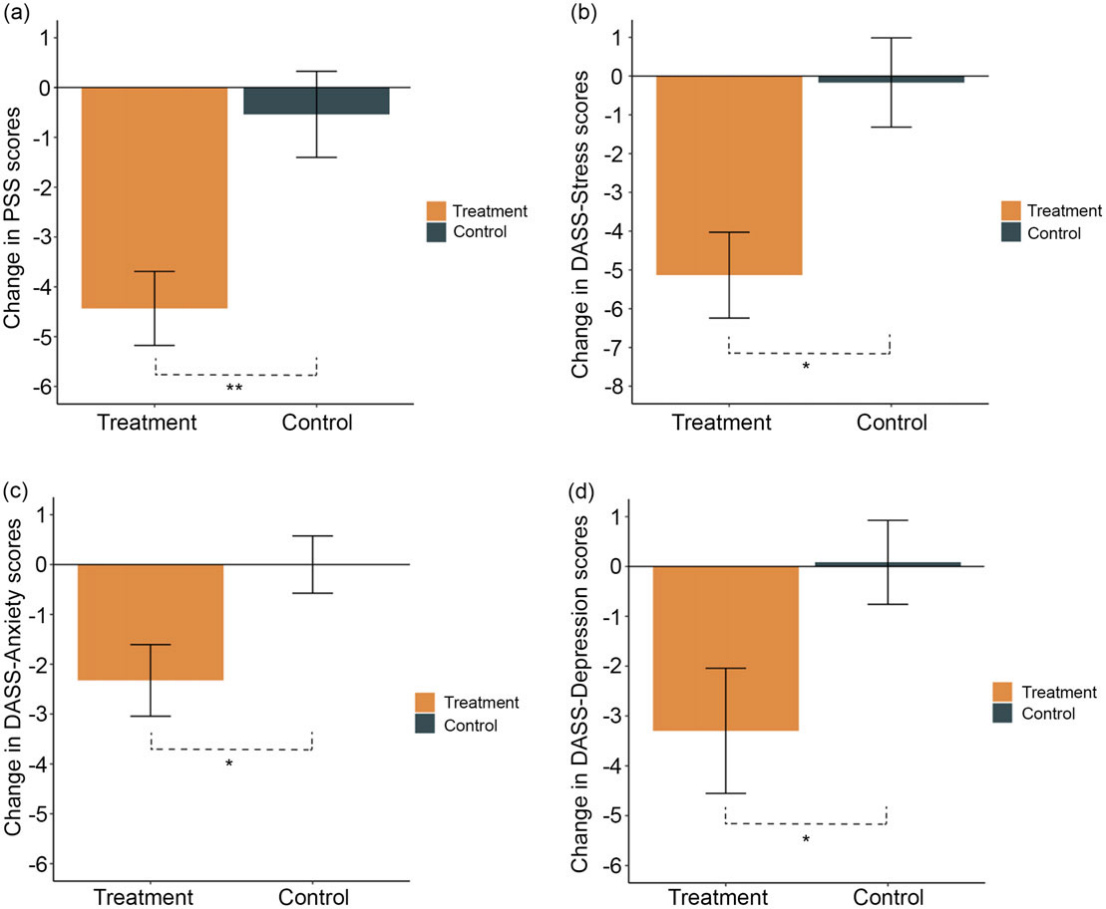
Changes from baseline to week 12 in (a) Perceived Stress Scale (PSS), (b) Depression, Anxiety, and Stress Scale 42-item (DASS) – Stress (DASS-S), (c) DASS – Anxiety (DASS-A), and (d) DASS-Depression (DASS-D) scores in the treatment (orange) and control (green) groups. Mean and standard errors are shown. * indicates *P* value < 0·05 and ** indicates *P* value < 0·005.

There was a significant reduction in perceived stress (PSS (*t*_*29·03*_ = −3·15, *P* = 0·004, *d* = 0·88)), stress (DASS-S (*t*_*29·13*_ = −2·81, *P* = 0·01, *d* = 0·76)), anxiety (DASS-A (*t*_*38·4*_ = −2·31, *P* = 0·03, *d* = 0·57)) and depression (DASS-D (*t*_*43·64*_ = −2·10, *P* = 0·05, *d* = 0·46)) from baseline to week 12 in the treatment compared with control group (Fig. 3(a)–(d); Table 2).

### Gut microbiome profiles

We observed no significant change from baseline to week 12 between groups in Shannon diversity (*W* = 264, *P* = 0·46, *HLE* = 0·06) or richness (*W* = 232, *P* = 0·17, *HLE* = 3·5). We also found no significant main or interaction effects in beta diversity (Bray Curtis) (*F* = 1·65, *P* = 0·08, *perms* = 9999) (online Supplementary Fig. 2). Multivariate analyses using MaAsLin2 revealed significant increases in the genera *Bifidobacterium* (*p*_*FDR*_ = 0·007, *q* = 0·24) and *Parabacteroides* (*p*_*FDR*_ = 8·79 × 10^–5^, *q =* 0·003) in the treatment group following the prebiotic intervention, but no significant differences between groups at week 12. In our exploratory analysis including hs-CRP as a fixed effect, we found that higher abundance of *Lactococcus* (*p*_*FDR*_ = 0·001, *q =* 0·05)*, Veillonella* (*p*_*FDR*_ = 0·003, *q =* 0·15) and *Rothia* (*p*_*FDR*_ = 0·004, *q =* 0·21) was associated with higher hs-CRP.

### Blood pressure and lipid profiles

We observed a significant reduction in systolic BP from baseline to week 12 in the treatment group compared with control (*t*_*40·67*_ = −2·21, *P* = 0·03, *d* = 0·29). No significant changes were observed between groups for diastolic BP (*t*_*43·65*_ = 0·27, *P* = 0·79, *d* = 0·20) total cholesterol (*t*_*14·85*_ = 0·37, *P* = 0·72, *d* = 0·75), TAG (*t*_*18·34*_ = 1·71, *P* = 0·11, *d* = 0·15), LDL-cholesterol (*t*_*14·86*_ = 0·44, *P* = 0·67, *d* = 0·18), or HDL-cholesterol (*t*_*24·91*_ = −0·51, *P* = 0·62, *d* = 0·12) (Table 2).

### Exploratory analysis examining associations between gastrointestinal symptoms, high sensitivity C-reactive protein and affective symptoms

**Fig. 4.**
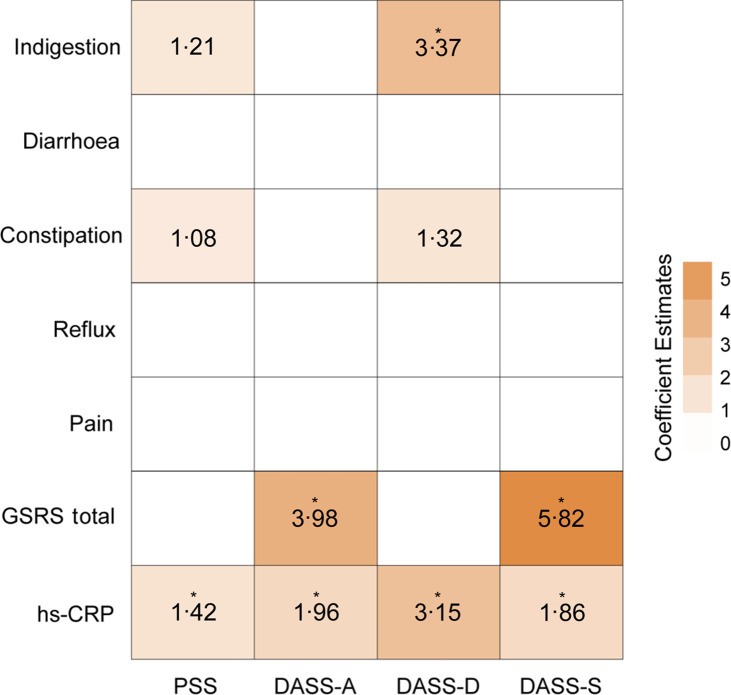
Heatmap showing multiple regression coefficient estimates representing the association between affective scores, with gastrointestinal and inflammation measures. Each column represents a different multiple regression model. PSS, Perceived Stress Scale; DASS, Depression, Anxiety, and Stress Scale 42-item; DASS-A, DASS-Anxiety; DASS-D, DASS-Depression; DASS-S, DASS-Stress; GGSRS, Gastrointestinal Symptom Rating Scale; hs-CRP, high sensitivity C-reactive protein. * indicates *P* < 0·05 (Bonferroni corrected).

For this exploratory analysis, we pooled together all participants (adjusting for the effects of repeated measures) and performed four forward stepwise linear regression analyses. Our results showed significant multivariate associations between gut-related and inflammatory markers with higher scores on all four psychological scales (Table 3). Specifically, PSS scores were positively associated with hs-CRP (*β* = 1·42, *t*_(86)_ = 2·39, *P* = 0·019), DASS-S scores were positively associated with hs-CRP (*β* = 1·86, *t*_(88)_ = 2·22, *P* = 0·029) and GSRS (combined score) (*β* = 5·82, *t*_(88)_ = 4·59, *P* = 2·09 × 10^–5^), DASS-D scores were positively associated with hs-CRP (*β* = 3·15, *t*_(87)_ = 4·20, *P* = 6·39 × 10^–5^) and GSRS (indigestion) (*β* = 3·37, *t*_(87)_ = 4·05, *P* = 0·0001) and DASS-A scores were positively associated with hs-CRP (*β* = 1·96, *t*_(88)_ = 4·66, *P* = 1·14 × 10^–5^) and GSRS (combined score) (*β* = 3·98, *t*_(88)_ = 6·15, *P* = 2·19 × 10^–8^) (Fig. 4 & Table 3).

**Table 3.** Results from four multiple regression analyses showed a significant association between gastrointestinal symptoms and hs-CRP on perceived stress (PSS) and depression, anxiety and stress scales (DASS-42)

Model	F-statistic	R^2^	Adjusted R^2^	*P* values
PSS	6·09	0·22	0·18	**0·001**
DASS-A	28·14	0·39	0·38	**2·13 × 10** ^**–9**^
DASS-D	16·97	0·37	0·35	**5·52 × 10** ^**–8**^
DASS-S	12·01	0·21	0·20	**0·0001**

PSS, Perceived Stress Scale; DASS, Depression, Anxiety, and Stress Scale 42-item; DASS-S, (DASS)-Stress; DASS-A, DASS-Anxiety; DASS-D, DASS-Depression. All *P* values are Bonferroni-corrected.

The bold values are statistically significant.

## Discussion

Our study showed significant changes in inflammation, the gut microbiome, and affective symptoms following a 12-week prebiotic fibre intervention with healthy diet advice in MetS, relative to participating receiving dietary advice only. Our primary outcome, hs-CRP, exhibited a significant reduction from baseline to week 12 in the treatment group relative to the control. This reduction is consistent with two independent meta-analyses in overweight and obesity, showing reductions in circulating hs-CRP following a fibre-rich food intervention or supplement^(56,57)^. Another meta-analysis combining participants with underlying inflammatory conditions, including type 2 diabetes, hypercholesterolemia, liver disease, inflammatory bowel diseases, and obesity, showed similar decreases in hs-CRP following prebiotic oligosaccharides compared with control^(58)^. Recent work by our group observed larger reductions in hs-CRP when using a similar combination of prebiotic fibres in pre-diabetes^(32)^. The discrepancy between this work and our current study could, in part, be explained by a larger daily dose (20 g), a longer intervention duration (16 weeks), or higher baseline hs-CRP levels. Specifically, we observed a considerable degree of variability in baseline hs-CRP levels, which may reflect heterogeneity in terms of the type, number and severity of MetS symptoms. Despite large inter-individual variability, our findings confirm the presence of low-grade systemic inflammation in many individuals with MetS, which may be improved through a diverse prebiotic fibre intervention.

We observed significant improvements in the PSS, DASS-S, DASS-D and DASS-A in the treatment compared with the control. While our affective outcomes were based on sub-clinical (i.e. no diagnosed neuropsychiatric disorders) scales, we also observed that the GAD and PHQ scales showed reductions in the treatment group too. Specifically, the treatment group shifted from the mild to minimal anxiety bracket (Generalised Anxiety Disorder (7-item)) and from the mild to minimal depression bracket (Patient Health Questionnaire-9). Consistent with these findings, a number of recent studies have shown improvements in affective symptoms following prebiotic interventions^(24–29)^, often accompanied by increases in *Bifidobacterium* ^(24,26,29)^. In this study, we too observed significant increases in *Bifidobacterium* and *Parabacteroides* in the treatment group, both of which have been linked to fibre degradation and the subsequent production of SCFA^(59,60)^. It is important to note that we did not observe any between-group differences in these genera at week 12. This may in part be attributed to the larger sample size in the treatment group compared with control, which increased our power to detect within-group differences. Contrary to what we expected, our exploratory gut microbiota analysis did not observe a significant association between greater abundance of *Bifidobacterium* and *Parabacteroides* with lower hs-CRP values. However, we did find that higher abundance of *Lactococcus, Veillonella and Rothia* was associated with increased hs-CRP levels, suggesting a potential pro-inflammatory role. However, there remains limited evidence for the role of these genera in the context of MetS, so care should be taken when interpreting these findings. SCFA production is thought to be one of the main mechanisms driving the relationship between prebiotic fibre intake and improvements in brain activity and affect. As part of this mechanism, SCFA downregulate systemic inflammation by maintaining intestinal barrier integrity^(61,62)^, promoting mucous production^(63)^ and regulating the secretion of interleukins^(18,64)^. This is particularly relevant here, as pro-inflammatory cytokines can influence neuroinflammation and associated changes in brain activity and behaviour by crossing the blood–brain barier or interacting with the blood–brain barrier interface^(8,14)^. SCFA also signal to the brain via enteroendocrine-mediated vagal signalling, offering a fast, direct and accessible route to influence mood, stress and anxiety^(19,20)^. As a complementary mechanism, SCFA have been shown to attenuate the stress response acting via the hypothalamic–pituitary–adrenal axis and may modulate the relationship between prebiotic fibre and perceived stress^(65,66)^.

Providing preliminary support for the above, our work suggests that systemic inflammation may be closely associated with mood and affective scores. Specifically, results from our exploratory stepwise multiple regression analyses showed that higher hs-CRP and GI symptoms were associated with higher scores of depression, anxiety and stress. The coefficient estimate for hs-CRP was strongest in depression, where changes in hs-CRP and GSRS (total) explained 35 % of the variability in DASS-D scores. The strength of this effect is consistent with previous work assessing inflammatory cytokines with depressive symptoms^(67,68)^, and functional connectivity changes in depression-related brain networks^(69,70)^. More broadly, this finding is in line with the cytokine-depression response, where acute inflammatory responses can manifest as social avoidance, anhedonia, fatigue and depressed mood^(9–11)^. The contribution of GI symptoms (GSRS) to depression, anxiety, and stress scores were also a consistent feature across regression models. GI symptoms have been recognised as a risk factor for poor mental health. That is, while constipation is generally considered a sequalae of depression or a side-effect from anti-depressant medication, a recent study shows it may be an independent risk factor or a prodromal symptom of depression^(71)^. Taken together, our results suggest two overlapping routes via which peripherally related signals may influence mood and affect: by regulating inflammation, and/or directly and indirectly improving GI symptoms.

The strengths of our investigation include the randomised controlled trial design and high tolerability to the intervention. However, several caveats need to be considered. As a pilot study, our sample size is relatively small and limited by the unbalanced randomisation allocation. While this provided necessary insights about the tolerability and side effects from using the prebiotic blend, a future extension of this work should involve a larger, placebo-controlled and balanced study design. Another consideration is that our control group did not take a placebo, which limits us from identifying placebo effects from taking the prebiotic blend. However, this is the first study to show multivariate associations between biologically relevant markers, including the gut microbiome and hs-CRP, with depression, stress and anxiety. This has enabled us to see considerable consistency with previous observational and interventional studies. Over the 12-week intervention, average adherence to the intervention was 73·0 (28·5) % (mean (sd)). The two predominant reasons for non-compliance were due to forgetting to take it, or travel disrupting their normal routine. While participants were advised to consume the supplement alongside an already well-established routine (e.g. adding it to morning coffee), future work should focus on additional strategies that optimise habit formation, including push notifications or alarm reminders on phones. Given that adherence rates could be improved, our results can be interpreted as relatively conservative, whereby higher adherence may strengthen our already significant findings. In addition to boosting adherence, this study may also benefit from providing participants with pre-measured single-dose sachets instead of the custom-made 10 g scoop. This would ensure that daily doses did not fluctuate between participants or across the study duration. Another limitation is that while the treatment and control groups did not statistically differ in baseline characteristics, the control group reported more GI and depression scores, hence, our findings should be interpreted with caution. We also cannot exclude the possibility that increasing prebiotic fibre intake could have led to increases in satiety, reduced caloric intake and subsequent weight loss, which may drive lower inflammation. However, results from our anthropometric endpoints did not show significant changes between groups from baseline to week 12, suggesting that changes in hs-CRP do not appear to be driven by anthropometric changes. While our study assessed dietary fibre intake at baseline and week 12, future work could benefit from incorporating additional dietary recalls and adherence monitoring tools to provide a more comprehensive evaluation of adherence to the UK Heart dietary guidelines and the role of other nutrients in improving metabolic and mental health outcomes. While it is highly plausible that the production of SCFA influenced the observed changes in primary and secondary outcomes, a further limitation in this study is that these metabolites were not quantified due to due to logistical constraints. In order to accurately quantify fecal SCFA concentrations, samples have to be immediately processed and flash frozen at −80°C due to high SCFA volatility, which was prohibitive for the participants due to the de-centralised nature of this randomised controlled trial. Moreover, SCFA are continually produced in and absorbed by the colonic epithelium, limiting the accuracy of stool samples in assessing the total SCFA exposure of a patient; thus, future studies would likely benefit from measuring circulating SCFA as they provide a rigorous snapshot of prebiotic fermentation^(65,72)^. Future work should also explore other inflammatory biomarkers beyond hs-CRP, due to different dynamics and timescales observed across inflammatory cytokines.

### Conclusion

Underlying systemic inflammation has been shown to contribute to alterations in brain activity, behaviour and affect. As such, there is a growing interest to develop novel, simple and cost-effective interventions that can target systemic inflammation to improve long-term psychological health. Here, we showed that a 12-week long daily prebiotic fibre intervention lowered inflammation, modulated gut microbiome composition and reduced sub-clinical levels of stress, anxiety and depression in MetS. Larger and placebo controlled randomised controlled trials (controlling for various participant and sample characteristics)^(73)^ will be necessary to confirm these findings and explore the mechanisms that impact the gut microbiota, inflammation (ideally by using wide range of inflammation biomarkers measured at multiple time points to control for intra-individual variations) and the brain.

## Supporting information

Hall et al. supplementary material Hall et al. supplementary material

## Supplementary material

For supplementary material/s referred to in this article, please visit https://doi.org/10.1017/S0007114524002186
